# Artificial Intelligence and Its Role in Diagnosing Heart Failure: A Narrative Review

**DOI:** 10.7759/cureus.59661

**Published:** 2024-05-05

**Authors:** Diptiman Medhi, Sushmitha Reddy Kamidi, Kannuru Paparaju Mamatha Sree, Shifa Shaikh, Shanida Rasheed, Abdul Hakeem Thengu Murichathil, Zahra Nazir

**Affiliations:** 1 Internal Medicine, Gauhati Medical College and Hospital, Guwahati, Guwahati, IND; 2 College of Medicine, Chalmeda Anand Rao Institute of Medical Sciences, Karimnagar, IND; 3 Internal Medicine, Sri Venkateshwaraa Medical College, Tirupati, IND; 4 Cardiology, SMBT Institute of Medical Sciences and Research Centre, Igatpuri, IND; 5 Emergency Medicine, East Sussex Healthcare NHS Trust, Eastbourne, GBR; 6 General Internal Medicine, Royal Sussex County Hospital, Brighton, GBR; 7 Internal Medicine, Combined Military Hospital, Quetta, Quetta, PAK

**Keywords:** smart watches, ecg interpretation, machine learning (ml), heart failure, artificial intelligence in medicine

## Abstract

Heart failure (HF) is prevalent globally. It is a dynamic disease with varying definitions and classifications due to multiple pathophysiologies and etiologies. The diagnosis, clinical staging, and treatment of HF become complex and subjective, impacting patient prognosis and mortality. Technological advancements, like artificial intelligence (AI), have been significant roleplays in medicine and are increasingly used in cardiovascular medicine to transform drug discovery, clinical care, risk prediction, diagnosis, and treatment. Medical and surgical interventions specific to HF patients rely significantly on early identification of HF. Hospitalization and treatment costs for HF are high, with readmissions increasing the burden. AI can help improve diagnostic accuracy by recognizing patterns and using them in multiple areas of HF management. AI has shown promise in offering early detection and precise diagnoses with the help of ECG analysis, advanced cardiac imaging, leveraging biomarkers, and cardiopulmonary stress testing. However, its challenges include data access, model interpretability, ethical concerns, and generalizability across diverse populations. Despite these ongoing efforts to refine AI models, it suggests a promising future for HF diagnosis. After applying exclusion and inclusion criteria, we searched for data available on PubMed, Google Scholar, and the Cochrane Library and found 150 relevant papers. This review focuses on AI's significant contribution to HF diagnosis in recent years, drastically altering HF treatment and outcomes.

## Introduction and background

Heart failure (HF) continues to be a significant health issue globally, with an ongoing trend of an annual rise in its prevalence in the USA by 2030 [1,2]. It is a condition in which the heart fails to pump enough blood per the body's requirements [3]. It is a dynamic disease with a changing trajectory of various syndrome attributes ranging from the pathophysiological processes to the disease progression [4]. It can also be understood as a clinical syndrome with varying definitions or classifications due to multiple pathophysiologies and etiologies [4]. Consequently, the diagnosis, clinical staging, and treatment of the syndrome become complex and subjective in clinical settings, which has implications for the prognosis and mortality of the patients [4].

With technological advancements, it cannot be denied that there is a parallel rise in the importance of their application in medicine [5]. Artificial intelligence (AI) is one example, and its role in medicine is currently being explored [5,6]. It can be used for tasks requiring human intelligence [6]. Although it is commonly used in smart devices to perform tasks, like automating and assisting people in translation, web browsing, filling sentences, or correcting words, it has excellent potential for its use in cardiology [7].

Machine learning (ML), or AI, involves the process of learning, where it familiarizes itself with a large set of data and identifies and interprets patterns better than a human brain [6-9]. ML has one of the techniques called deep learning (DL). It uses neural networks to read, analyze, and interpret large and complex data collections [5,6,10]. In outpatient department (OPD) settings, it is not easy to diagnose HF in its early stages [11]. First, the clinical presentation of HF often has non-specific symptoms, like cough or dyspnea, which can be misinterpreted as pulmonary disorders and, hence, cause a delay in diagnosing HF [11]. Second, expensive tests like echocardiography, exercise stress tests, and natriuretic peptides are not readily available in low-resource setups [11]. However, using the assistance of AI in ECG and X-ray imaging, which are inexpensive and non-invasive investigations done early to diagnose HF and other cardiological abnormalities, can result in the early detection of HF [11,12]. 

The vast number of variables and quantity of data required to process and diagnose a complex syndrome like HF is a challenge for the human brain [10,13,14]. Thus, the ability of AI to identify and interlink subtle findings is an asset in efficient and effective decision-making [10,13,14]. Gladding et al. [14] incorporated AI with multiple sources of information from patients suffering from HF [14]. It included sources like DNA sequencing, genomics, metabolites, data from wearables, digital ECG findings, and echocardiography [14]. These data were used to substratify HF patients and show AI's potential to diagnose HF compared to conventional techniques [14]. Meanwhile, Kotanidis et al. [10] demonstrated the use of AI in screening a population to detect coronary artery disease (CAD) in the community and thus provide guidance in further testing and diagnosis [10].

**Figure 1:**
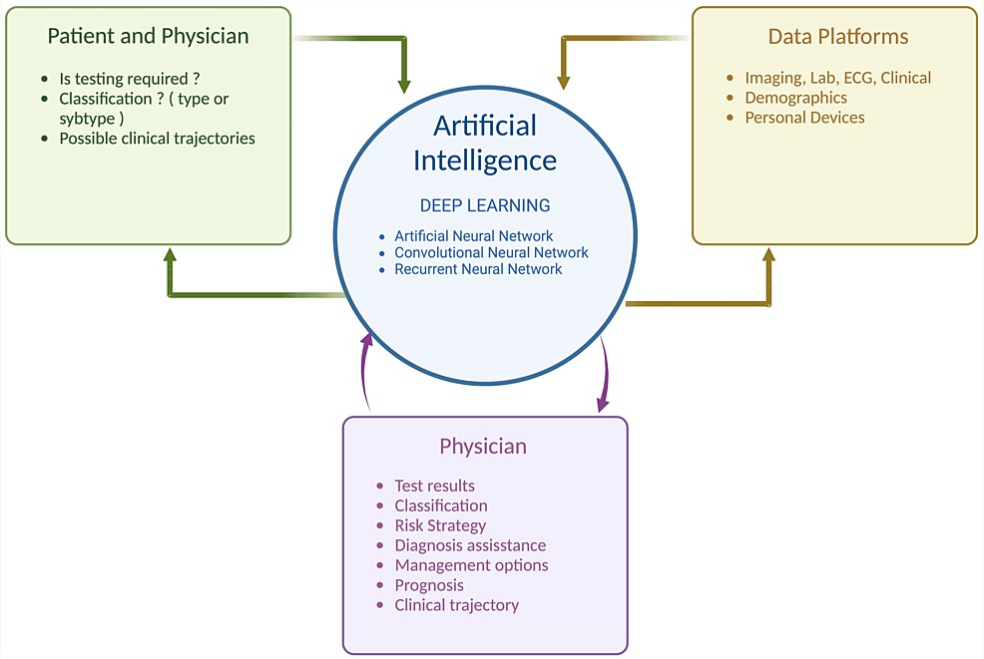
Potential flow of input and output of data between various sources to the incorporated artificial intelligence system Source reference: [5,6,10]

In this study, we aim to discuss AI juxtaposed with conventional diagnostic methodologies for HF, scrutinize the amalgamation of AI for early detection of HF, elucidate its potential for cost mitigation, delineate future applications inclusive of wearable technologies, and evaluate present limitations inherent in this paradigm. Figure 1 depicts the input and output of data from various sources incorporated into AI.

## Review

AI focuses on equipping machines with human intelligence and decision-making capabilities to offer forms of support to individuals [15]. The AI concept of learning can be broadly classified into ML and DL [16]. AI is a term that depicts machines to simulate human intelligence [17]. It revolves around training machines to make decisions independently through various algorithms and mathematical calculations without actual human involvement [18]. AI utilizes technology to provide personalized treatment plans for patients [19]. It excels at handling tasks that may be too complex or demanding for humans, showcasing its potential [20]. AI enhances physicians' performance in hospital environments. By leveraging computer systems tailored for this purpose, healthcare professionals can prioritize patients requiring immediate attention based on their physiological indicators [21]. However, a notable drawback of AI is its reliance on technology, which can incur development, maintenance, and repair costs. Furthermore, if misused or mishandled, AI-powered machines can cause disruptions [22]. ML contributes to a significant chunk of AI today. It involves training machines to learn from existing data and apply it to new data to identify similar patterns and produce a predictive output [17,18]. Based on how machines are trained to acquire and interpret data, ML is further subdivided into supervised, unsupervised, and reinforcement learning [16].

ML

Different types of AI exist within the realm of intelligence. ML plays a role in research and is an integral component of AI applications in various fields [23]. ML, an intelligence (AI) component, empowers computers to process data in ways beyond traditional programming methods. It involves identifying patterns in data, applying patterns to information, and surpassing human capabilities in computational tasks [24]. 

**Figure 2:**
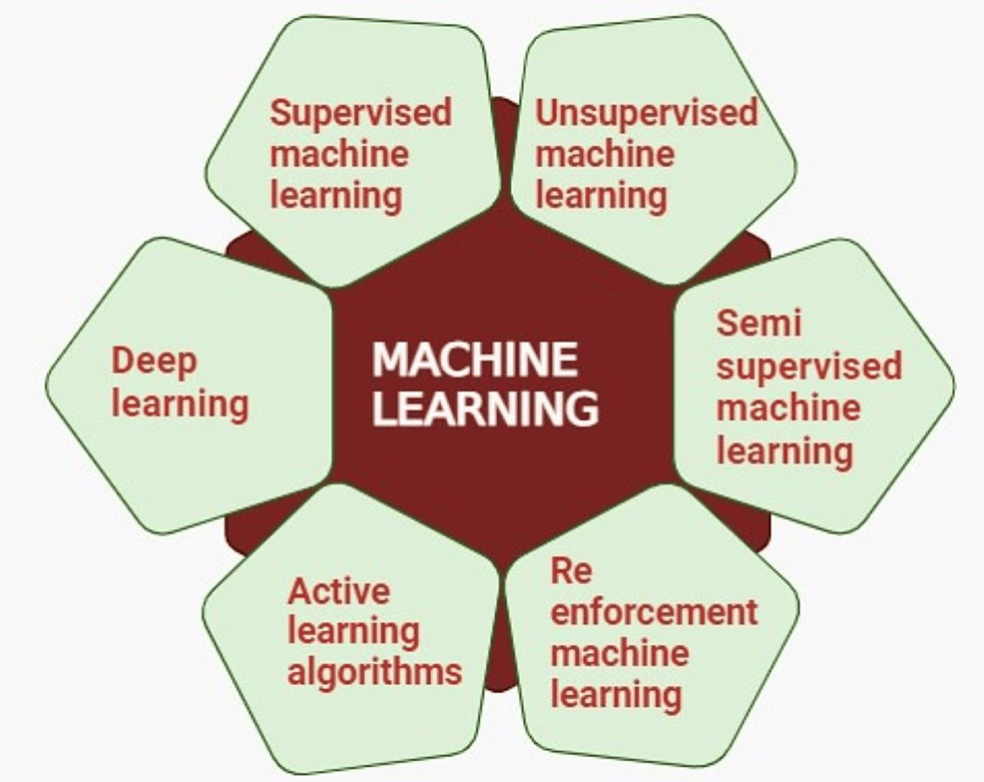
Machine learning and its various types Source reference: [25]

There are five categories within ML: supervised, semi-supervised, unsupervised, reinforcement, and active learning algorithms. DL techniques represent an advanced stage within ML that utilizes networks to classify data and produce more precise predictions [25]. Categories of ML are shown in Figure 2.

In supervised ML, human-labeled datasets guide algorithms to accurately categorize information or forecast outcomes [18]. This approach has successfully diagnosed HF using electrocardiography time series data [26]. Various algorithms, such as support vector machines (SVMs), artificial neural networks (ANNs), Bayesian networks, AdaBoost random forest techniques, and LASSO regression, are examples of ML methods [27].

**Figure 3:**
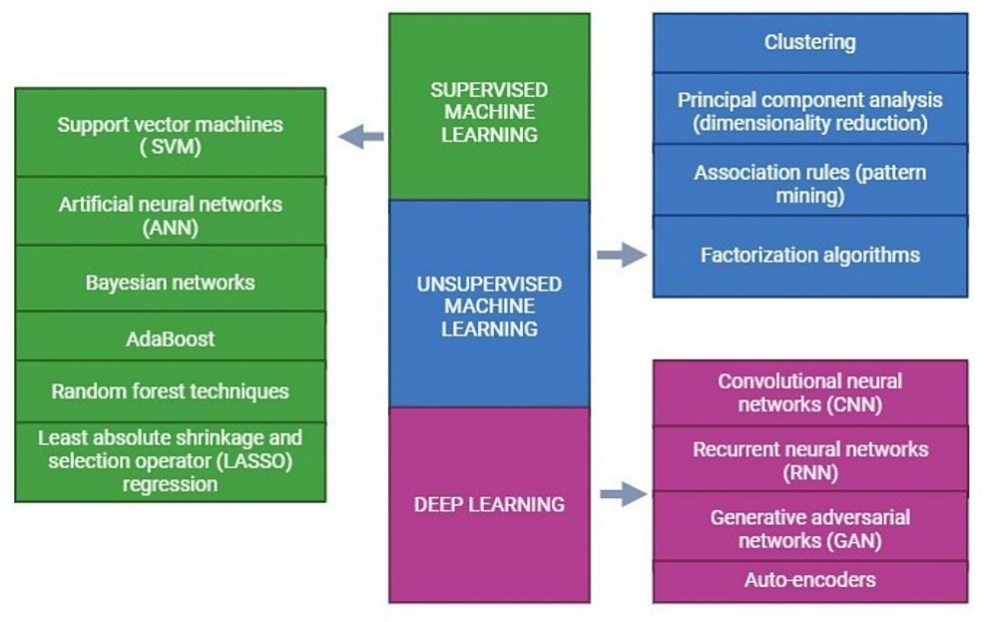
Components of machine learning and deep learning Source reference: [39,41,42]

Unsupervised ML involves grouping and analyzing unlabelled datasets to uncover hidden patterns without human intervention [18]. Unsupervised learning algorithms, such as clustering, principal components analysis (dimensionality reduction), association rules (pattern mining), and factorization algorithms, are commonly used in the healthcare field to identify patterns and relationships within data (Figure 3). Clustering, in particular, is widely utilized for this purpose [28]. Semi-supervised learning combines elements of both supervised and unsupervised approaches using labeled and unlabelled datasets. Unlabelled data are grouped based on similarities with labeled data to enhance the learning process [18]. Reinforcement learning, often used in robotics and gaming scenarios, relies on negative feedback to improve models. Machines with sensors, cameras, and GPS systems interact with their environment to better understand their surroundings [29].

DL

DL within AI/ML excels at handling intricate datasets derived from images or videos used in medical contexts. Neural networks, which consist of interconnected nodes known as neurons organized into input layers and an output layer, typically represent the structure of deep learning. When a trained neural network receives data, specific neurons in the input layer become active and transmit signals to neurons in the layers and eventually to the output layer. This process results in the refinement of information as it traverses through layers. DL algorithms have been widely utilized in medicine for supervised and unsupervised training to automate the analysis of images [30]. The "McCulloch-Pitts (MP) neuron" was developed by neuroscientist Dr. Warren McCulloch and computer scientist Walter Pitts in 1943, and it functions analogously to the brain [31]. DL involves more hidden layers than traditional neural networks, enabling it to more effectively explore complex nonlinear patterns within data. With the growth in data volume and complexity in imaging analysis, the adoption of DL techniques in medical research has experienced a surge in popularity recently [32]. 

Convolutional neural networks (CNNs)

CNNs are a type of network widely used in computer vision, speech recognition, and facial recognition applications due to their minimal data pre-processing requirements [33]. The design of CNNs draws inspiration from the neurons in human and animal brains. They consist of stacks of convolution and pooling layers followed by fully connected and normalization layers. In CNN architecture, the convolutional layer plays a crucial role. CNN can process both 2D and 3D images, reduce complexity, and automatically extract features, eliminating the need for labor techniques. This is made possible by their structure. Critical characteristics of CNNs include weight sharing (adjacent neurons sharing weights) and local connectivity (neurons in one layer connected to neurons in the next layer focusing on essential information). These features speed up the training process of CNNs with human involvement [34,35], initially advocated for CNNs in analyzing dimensional images, making them one of the most commonly used DL networks [36]. In CNNs, normalized pixel values of images are used as inputs for processing. Through subsampling in layers and weighted convolutions, CNNs transform values into a series of weighted input values through a recursive function [32].

Recurrent neural networks (RNNs)

In RNNs, outputs from steps are fed as inputs to subsequent layers in each step. One of its characteristics is a state that retains sequence details and functions as a memory component. This state remembers past inputs, simplifying the parameter complexity compared to networks by using the same parameters for each input in its hidden layer, ensuring consistency in task performance [37].

RNN-based approaches have been introduced that can handle amounts of linear data effectively. These methods could be ideal for managing the data transmitted through monitoring systems. RNNs are commonly used in time series forecasting because, unlike networks that are strictly feed-forward, RNNs allow the output from hidden layers to loop back into neurons within the network. The final output is then generated by combining this state output with input data. The predicted output is compared to the ground truth during training to calculate errors. Subsequently, backpropagation is utilized to compute gradients and adjust layer weights for predictions. RNNs may encounter challenges, such as vanishing or exploding gradients during backpropagation, adversely impacting the models' performance. As a result, RNNs excel when provided with input; however, their accuracy diminishes as data volume increases. Gated recurrent unit (GRU) and long short-term memory (LSTM) models are known for their ability to retain sequences of data through the use of "gates" addressing the issue effectively [38].

Generative adversarial networks (GANs)

GANs consist of two networks where the generator network creates instances, which are then evaluated as real or fake by the discriminator network based on their resemblance to the original training dataset. Both networks are trained simultaneously until realistic examples are produced by the generator. GANs have found utility in healthcare for image and data generation from records [39]. One notable application involved using GANs to convert CMR images into computed tomography, enhancing the visualization of calcified formations that may be challenging to detect on CMR scans [40]. By supplementing missing data, GANs have improved predictive model performance, as demonstrated by Che et al. [41], who integrated GAN-generated data with patient data to enhance a CNN-based risk prediction model [41]. Using algorithms like GANs and variational autoencoders has proven effective in generating synthetic medical images, such as X-rays, CT scans, and MRIs. These technologies play a role in enhancing the accuracy and robustness of medical imaging systems [42]. Figure 3 depicts the generative components of ML and DL.

AI clinical decision support system (CDSS)

An AI CDSS could aid in detecting HF in high-risk patients through AI algorithms. AI CDSS offers an adaptable medical assistant platform for medical conditions. AI CDSS comprises five layers: user interface management layer, knowledge acquisition and inferencing layer, context recognition and monitoring layer, data acquisition and persistence layer, and engineering support layer. The knowledge acquisition and inferencing layer as the layer creates knowledge models by combining expert-made rules with rules generated from data like text and images, continuously updating knowledge over time [18].

Choi et al. [43] developed an AI CDSS for HF diagnosis and evaluated its accuracy. It demonstrated a 98% accuracy for HF compared to non-specialists with 76% accuracy. This suggests that the AI CDSS could be beneficial in identifying HF cases, especially when access to specialized HF professionals is limited [43]. In addition, CDSS has been utilized in diagnosing, preventing, and managing chronic diseases. While facing challenges and limitations, the system provides a decision-making tool to enhance healthcare standards. AI CDSSs utilize ML and natural language processing (NLP) techniques to gather insights from unorganized data sources. Enhancing the system’s accuracy involves merging expert-driven knowledge acquisition with rule development powered by ML [44-49]. 

NLP involves extracting meaning from speech and text using various methods. These algorithms are developed through a combination of ML models, rules, or a blend of both approaches. In the field, NLP can be leveraged to extract clinical information from vast patient notes commonly found in electronic health (eHealth) records [50,51].

Traditional methods of diagnosing

In HF, there is a group of symptoms due to various circulatory and neurohormonal responses of the body to various cardiac insults in the presence of an objective cardiac dysfunction [4,52,53]. The intricacy of diagnosing HF lies in the fact that it can be caused by various disease pathologies, like ischemic heart disease, valvular heart disease, cardiomyopathies, or simply hypertension, each with a varying disease course, making it challenging and a demanding diagnosis to make [3,4,54]. Many interconnected factors make it one of the most expensive diseases to treat, even in developed countries [33]. Another study found an inverse relation between socioeconomic status and inflammatory biomarkers related to HF, which in turn could be a consequence of stress-related responses of the neurological, endocrine, and autonomic systems [55]. Furthermore, a decrease in utilization and access to healthcare with decreasing socioeconomic status is prevalent across the globe, including in countries with universal healthcare systems [55].

Currently, the diagnosis of HF is a composite process involving clinical history, signs and symptoms, and imaging techniques to assess ventricular functions and biomarkers for progression [53]. These procedures can be very expensive and cumbersome for cardiologists. Moreover, the limitations of existing traditional methodologies pose an additional risk of false positives and missed diagnoses [56]. 

Blood Tests

B-type natriuretic peptide (BNP) is a hormone mainly produced by the ventricles, and its plasma levels rise significantly in people with HF. Moreover, levels of BNP can also increase in conditions like heart attacks and when the heart's pumping function is weakened. BNP is produced in response to the heart being stretched and distended or due to neurohormonal activation. When the cardiomyocytes make BNP, a larger molecule called preproBNP is initially synthesized, which then gets split into two parts: active BNP and NT-proBNP, which is not active. BNP helps inhibit the renin-angiotensin mechanism and adrenergic activities and causes natriuresis and vasodilation. Hence, in conditions with an acute increase in the ventricular volume, this hormone plays a vital role in countering this by opposing vasoconstriction, retention of sodium, and the renin-angiotensin system [57].

However, there are limitations to using these biomarkers to diagnose HF [58,59]. These biomarkers are present at higher levels in HF patients than in healthy individuals. Blood levels of BNP and NT-proBNP have a high diagnostic accuracy in diagnosing HF in patients presenting with dyspnea and are therefore commonly used in this regard [58,59].

The presence of inflammation can alter the levels of these cardiac biomarkers [60]. The presence of concomitant inflammation in patients with HF can cause the levels of these peptides to be elevated, corresponding to the absence of such inflammation [60]. Trotter et al. [60] conducted three separate studies to understand the association between inflammation and NP (natriuretic peptides) levels, which showed that inflammation may be a trigger for NP release; therefore, the inflammatory conditions should be considered when interpreting NP levels [60].

Second, renal function affects the clearance of NT-proBNP, which leads patients with renal impairment to have elevated levels of NT-proBNP. Since the clearance mechanisms of BNP and NT-proBNP are different, this causes a difference in the levels of BNP and NT-proBNP. Furthermore, the presence of comorbidities, such as obesity, anemia, and AF, can also influence serum NT-proBNP levels [61].

Imaging

Imaging techniques are vital to the diagnosis of HF [62-64]. Even though the diagnosis of HF is done clinically, transthoracic echocardiography is the first line for confirmation of the diagnosis and classification [62,63]. It gives information about cardiac anatomy and hemodynamics [62]. Transthoracic echocardiography enables cardiologists to quantify the left ventricular ejection fraction (LVEF), which further helps in risk stratification of the patients and aids in patient management [62].

Other imaging techniques include X-rays, cardiac MRI, and computed tomography imaging (CT) [62,65]. Patients with HF commonly present with dyspnoea, in which case an X-ray is initially used to differentiate between pulmonary and cardiac causes [11]. Cardiac MRI has excellent utility in tissue characterization, which is helpful in various diseases, such as ventricle noncompaction and right ventricular cardiomyopathy [65]. On the other hand, CT imaging is functional for assessing coronary and valvular diseases and is also helpful in characterizing myopathies [65]. 

On the other hand, there are certain limitations to these traditional imaging techniques. The quality and interpretation of images can vary due to factors such as limited acoustic windows and dependency on the operator [65]. In addition, there are several other constraints in the use of imaging in HF, like patient-specific contraindications, accessibility, high cost, availability of technical expertise, and need for specialized centers [65-68].

Interventions of AI 

Even though advancements in Modern medicine over the last few decades have resulted in a decrease in the morbidity and mortality associated with almost all cardiovascular diseases, HF remains an exception [3]. Therefore, more advanced diagnostic modalities have become the need of the hour for early detection and treatment of HF [6].

With the advent of modern technologies, we now live in the "big data" era where we have enormous data in eHealth records, genomics, pathological results, imaging results, data from telemonitoring using implantables, wearable devices, and various mobile applications [18]. Hence, modern-day cardiologists need to assimilate these different data for early diagnosis and risk stratification, thus improving the quality of care in patients with HF and other cardiovascular diseases [30]. This explains the rising popularity of AI concepts of learning and its applications in cardiovascular medicine.

The scope of AI-guided big data analysis is enormous and yet to be fully explored [7]. These can potentially be used to predict the therapeutic efficacy of various treatments [69]. Moreover, this "big data" coupled with AI technologies can be further used to provide more individualized care to patients and simultaneously bring down costs [56,70].

Cost Reduction By the Integration of AI With Traditional Methods 

The risk of HF increased from 19.0% to 23.7% over 25 years between 1965-1989 and 1990-2014, according to the Framingham Heart Study (FHS) cohort [2]. Bozkurt et al. [2], depict a clear picture of how there Is not only a trend of increase in the prevalence of HF in the USA but also shows that there is an increased lifetime risk of HF of up to 24% [2]. Similarly, the HF prevalence, incidence, and prevalence of risk factors have increased globally [2]. A cohort study called PURE (Prospective Urban Rural Epidemiology) that involved more than 150,000 adults across low-income, middle-income, and high-income countries showed that the incidence of HF was similar across these countries despite a higher prevalence of risk factors in high-income countries [55]. It is a fact that the hospitalization and treatment costs for HF are very high, which is an existing problem. However, the burden of cost further increases when there are readmissions of patients [71]. HF patients have acute exacerbations that report high rates of hospitalization and involve a worldwide expenditure of around $31 billion yearly [29]. From the healthcare system perspective, the situation worsens when the shortage of healthcare workers comes into the picture [72]. On the other hand, for patients, the affordability of bearing the costs of a chronic disease like HF is not only high but the cost is further affected by the worsening quality of life and poor patient satisfaction [71,72]. There are several areas in cardiology where the incorporation of AI will help in the expenditure associated with the diagnosis of HF [43,69,73].

Enhanced Diagnostic Accuracy

HF patients have to undergo many tests, resulting in the collection of a vast amount of data in the records of the institutions. Rather than using traditional methods of analysis using human brains, it is far more feasible to use AI to do the job as it can recognize patterns and make use of them in multiple areas of HF management, eventually helping cut down expenses [74]. Also, this complex and sophisticated analysis of vast amounts of data by AI can help improve the diagnostic accuracy of these tests, which will not only directly save costs but also reduce the requirement for additional tests. However, it will also indirectly help cut expenses by reducing the diagnosis time, leading to early diagnosis and early initiation of treatment [6,18,32,43]. 

Choi et al. [43] created the Artificial Intelligence Clinical Decision Support System (AI-CDSS) and applied it in the diagnosis of HF among patients presenting with dyspnoea in the outpatient clinic. They used a hybrid system where ML was integrated with knowledge acquired by expert-driven acquisition, AI-CDSS demonstrated a higher diagnostic accuracy as compared to non-HF specialists [43]. This makes it a valuable tool for diagnosing HF, especially in the absence of specialists, thus cutting the costs of specialist consultations [43].

AI in reading ECGs 

Additionally, implementing AI with low-cost medical investigation tools, like ECG, in large populations will result in bigger cost savings [12,75]. In a study conducted by Bachtiger et al. [75], an ECG-enabled stethoscope and a deep learning system called AI-ECG (a CNN) were used, and it was found that the single-lead ECGs could be used to detect HFrEF with an LVEF of 40% or lower [75]. 

Another paper based on ECG changes by Garcia-Escobar et al. [12], mentions numerous subtle ECG changes corresponding to several heart conditions and discusses the importance and relevance of ECG as a cost-effective and accessible tool in cardiology with the application of AI. To elaborate, a few points discussed are - low QRS voltage may be associated with ischemic and non-ischaemic cardiomyopathies along with congestive HF, and small QT interval increases are associated with diastolic dysfunction, which arises from conditions like myocardial ischemia, hypertension and diabetes; and that in an ECG of a left ventricular branch block that also shows subtle changes in specific patterns like the QRS voltage and axis, the presence of additional conditions such as right ventricular dilatation can be detected [12]. In conjunction with this, it focuses on the point that there are significant practical implications of the use of AI for a more efficient and thorough evaluation of reading ECGs and that AI will speed up and enhance the diagnostic utility of ECGs in diagnosing heart conditions, therefore, reducing expenses by potentially eliminating the need of additional imaging and other expensive tests [12]. 

Use of AI in automated analysis of imaging

On the other hand, the symptoms of HFpEF can be neither apparent nor specific; consequently, X-rays are widely used as the first and, at the same time, inexpensive imaging modality to differentiate the cardiac cause from the pulmonary cause [11]. A study used AI to interpret Chest X-rays for detecting HFpEF, which helps reduce the dependence on more expensive imaging modalities like echocardiography and cardiac MRI [11]. 

To re-iterate, early diagnosis of HF will lead to early treatment initiation and better chances of avoiding admissions and readmissions of patients, enabling better prognosis, lower cost-bearing, and better resource prioritization [75]. Thus, in the future, the application of AI to diagnose HF will not only give clinicians the ability to make precise decisions but also play a significant role in making the process more cost-efficient for the healthcare system and the patients [16,33,72,76]. 

AI in wearables 

The combination of wearable devices and AI can be used for cardiovascular monitoring at a fraction of the cost of medical-grade devices, which are very expensive. This results in more cost-efficient prevention and early detection of HF [77,78]. Moreover, having a large number of data for AI to assess is beneficial since it provides more examples for the learning process [77]. A study by Arman et al. [77] involves monitoring cardiovascular biomarkers, including heart rate and step count. It was achieved using consumer-grade smartwatches, cloud technology to store big data, and applying self-supervised and multiple-instance learning techniques with AI to detect HF and AF [77]. This demonstrates AI's potential to help facilitate cost-effective cardio-vascular monitoring of outpatients and prevent exacerbations in HF patients [77].

Classification costs

When HF is diagnosed and classified using traditional support systems for medical decision-making, the cost of false negative results is much higher than that of false positive results [79].

Besides this, the traditional systems do not consider the unequal misclassification costs between different categories [79]. Qi Zhenya et al. [79], used ML. They proposed an alternative tool in the form of an ensemble that offered better classification accuracy of HF patients and a reduced misclassification cost [79]. Moreover, complex algorithms and AI-based approaches in the future will produce more optimized ensemble methods to achieve better results with lower costs [79].

Readmission costs

In the context of rehospitalizations of HF patients, there is potential for the use of AI to evaluate the risk of readmissions of patients, thereby promoting the initiation of personalized and cost-efficient treatment [80]. Baashar et al. [80], have discussed studies that involve supervised ML, where labeled data are used as the input, and relationships between the given data and the label are determined to develop algorithms helpful in predicting the occurrence of an outcome [80]. For example, one of the studies used multiple algorithms to predict the readmission of more than 50,000 HF patients and compared their performance [80]. Another study, involving a meta-analysis of 20, found that the AI models performed better in terms of discrimination and accuracy of mortality prediction and rehospitalization of HF patients than conventional statistical models [80]. 

Furthermore, for risk stratification, unsupervised ML can be utilized to generate models that can identify patterns without labeling inputs. Another study hierarchically stratified HF patients with HFrEF (HF with reduced ejection fraction) along with mildly reduced ejection fraction and controls, which were linked based on several characteristics, and looked for differences in the level of the urinary proteome [80,81]. The study found remarkable differences in several urinary peptides between HF patients and controls, demonstrating their specificity for HF [80,81].

In summary, the cost of diagnosing and managing HF is influenced by several factors. However, it has a significant and close relation with the early diagnosis of HF and preventing disease progression. 

The following section provides a more comprehensive discussion and examples of integrating AI alongside traditional methodologies to enhance early detection capabilities for diagnosing HF.

Modalities for the early detection of HF

Early detection, diagnosis, and treatment are significant for HF, slowing its progression to the advanced stage and improving overall outcomes.

Tracking/Monitoring Basic Modalities 

To highlight once more, in the era of enormous data collection and available information, AI can revolutionize data organization and analysis, accurately predicting future observations and fast-tracking results [82]. With the ability to scale and process large sample sizes and the associated clinical data, AI creates relevant correlations, identifies patterns within the data, and generates specific outcomes. This reduces the time and effort required for manual data management and helps identify hidden patterns that may not be apparent to human analysts [82].

More deep-learning techniques are now used by researchers in clinical applications. One study by Lasko et al. [83] applied autoencoders to learn patterns from serum uric acid. Che et al. [84] discovered physiological patterns linked to recognized clinical characteristics using deep neural networks with incremental learning on clinical time series data. Lipton et al. [85] tracked pediatric ICU time series data like heart rate, blood pressure, glucose level, etc., using an extended short-term memory network (LSTM), a type of RNN for the prediction of multilabel diagnosis.

Tomov et al. [86] discovered a five-layer DNN model- named Heart Evaluation for Algorithmic Risk-reduction and Optimization Five (HEARO-5), which has high yield accuracy ( 99% accuracy and 0.98 MCC) in the prediction of heart disease-based on routine clinical data. Choi et al. [87] used GRU deep learning methods, a novel predictive model framework for diagnosing HF. Both traditional statistical methods (TSM) and ML have several distinct and overlapping characteristics when tracking primary modalities in HF [18]. Traditional statistical methods primarily rely on predefined mathematical models and assumptions. By contrast, ML algorithms can learn patterns directly from the data. This helps ML algorithms to adapt and improvise their predictions and results over time, making them well-suited for tracking primary modalities in HF [88]. 

ML models like random forests, decision trees, logistic regression, and support vector machines help interpret complex healthcare data and accurately track primary modalities associated with HFs, such as heart rate, blood pressure, ECG signals, cholesterol levels, classification and severity of HF, predicting adverse events like destabilization, rehospitalization, and mortality [30,89]. Healthcare providers can also gather these real-time data from wearable devices and mobile apps using AI algorithms [90]. The multi-variable statistical analysis from the data forms scores used in clinical practice, estimating mortality risk, rehospitalization, and morbidity like HF Survival Score, Seattle Heart Failure Model, and EFFECT [89]. Real-time patient status monitoring through AI integration with telemedicine and mobile health technology will allow for prompt treatments and prevent the development of adverse effects [29]. 

Leveraging AI and cardiac biomarkers 

Integrating ML algorithms and biomarker analysis can help healthcare professionals gain valuable insights into the underlying mechanisms of HF, identify high-risk individuals, and develop targeted early intervention strategies to prevent the progression of HF and potentially reduce mortality rates [91].

B-type and N-terminal pro-B-type natriuretic peptides (BNP and NT-proBNP, respectively) are recognized as highly significant diagnostic and prognostic indicators in cases of HF, among other cardiac biomarkers [57,92-95]. Novel techniques for identifying patterns in cardiac biomarkers are being developed by utilizing several machine-learning algorithms, such as clustering and phenomapping. Based on their protein biomarkers, Woolley et al. [96] reported that unsupervised cluster analysis on a wide range of circulating biomarkers helped identify clusters of heterogeneous patients with HF with preserved ejection fraction (HFpEF). 

Another study by Shah et al. [97] found that risk could be discriminated more accurately using phenomapping or unbiased cluster analysis of dense phenotypic data than NT-proBNP and risk scores alone. In addition, phenomapping has the potential to provide valuable insights for the planning and execution of upcoming clinical studies. It can also be utilized to identify individuals likely to react favorably to therapies, thereby addressing the persistently low success rate of clinical trials for HFpEF [97].

Shah et al. [97] also described support vector machines (SVM), a machine algorithm that identifies a separation boundary between classes of interest in a much higher-dimensional feature space [97]. Furthermore, the outcomes of these analyses can be applied to clinical trials to ascertain whether specific patient groups respond better to the investigational medication or device than other patient groups. This information can help improve future HF clinical trials and "theranostics," a combined therapeutic and diagnostic treatment approach [97].

Takefuji et al. [98] developed a universal app, phope.py (Patient for Hospital Observation and Predicting Effects of Medication and Exercise), that utilizes linear regression, which can be predicted with smaller datasets than conventional ML techniques. With five determinants, including "day" (test date), "hgb" of hbA1c value (diabetic biomarker), "BNP" of NT-proBNP value (HF biomarker), "degree1" (polynomial regression for hbA1c), and "degree2" (polynomial regression for NT-proBNP), phope.py is a versatile biomarker prediction tool designed for patients [98]. According to the author, 5668 people have used the Phope app worldwide [98].

Enhancement of cardiopulmonary stress testing in HF 

Cardiac stress tests are frequently used to assess the heart's functionality and efficiency. However, the interpretation of these tests can be arbitrary and may vary depending on the experience and expertise of the healthcare professional. One of the primary signs of HF is exercise-induced dyspnea and exhaustion [99]. Cardiopulmonary exercise testing, or CPX, has become a significant non-invasive method for monitoring the cardiopulmonary vitals of patients. Interpreting various time series variables from CPX requires significant time and effort. Comprehensive knowledge and a thorough comprehension of all variables, tables, and flow charts are necessary for the interpretation. However, the applications of CPX have increased due to recent advances in ML research. This multivariate time series problem of interpreting CPX involves simultaneously assessing gas exchange (oxygen uptake), carbon dioxide output, ventilation, and generated heart rate. These time series' manual evaluations and conventional analytics are reduced to peak values, summary indices, and slopes. The gold standard for evaluating cardiorespiratory fitness is the VO2 peak. In ML, this process of simplifying and extracting signals from lengthy time series is called feature engineering [100].

SVM, an ML tool, was used by Inbar et al. [101] to identify patients with chronic HF using CPX. Furthermore, because CPX testing involves rigorous testing, research is actively underway to develop ML-based methods for estimating patient response to exercise in the early stages, as well as for estimating the dynamics of oxygen consumption from heart rate and inputs from the treadmill, cycle, and accelerometer. These methods will be integrated into smart devices [100,102-104].

Myers et al. [105] showed that ANN outperformed traditional survival analysis on CPX data in assessing the risk of cardiovascular mortality. Sharma et al. [100] distinguished between patients with HF and those with metabolic syndrome using the strong performance of a small CNN on CPX data. 

Tabassin et al. [106] used an unsupervised statistical method and supervised classifier to model spatiotemporal variations of Left ventricular strain rate during rest and exercise to identify patients with HFpEF, where the diagnosis of HFpEF is challenging for healthcare professionals because ejection fraction is usual and cardiac congestion is problematic to evaluate non-invasively, also as many patients have hemodynamic abnormalities only during exercise [107-110].

Advanced cardiac imaging 

Multiple data sources, such as the ECG, EHRs, and imaging data (like echocardiography and cardiac magnetic resonance imaging), are combined to detect and diagnose HF. Newer AI algorithms are being developed to improve diagnostic accuracy further. Research includes echocardiography image analysis, natural language processing for EHR data mining, and ECG analysis [18]. Cho et al. [111] demonstrated that HF with a reduced ejection fraction could be screened by a wearable device that used AI algorithms and a single-lead ECG instead of a standard 12-lead ECG. By incorporating such algorithms, AI can significantly aid in analyzing raw imaging data from cardiac imaging methods [6,18]. AI implies mimicking human intelligence through computing techniques. Large medical datasets are often utilized in the healthcare industry to advise treatment plans, find new disease genotypes or phenotypes, or anticipate a diagnosis. Noninvasive imaging is still essential for diagnosis, treatment, and risk assessment of individuals with cardiovascular disease [73]. Echocardiography remains the most widely used cardiac imaging modality. The quick, point-of-care assessment of cardiac anatomy, function, and hemodynamics has been made possible by the growing use of handheld ultrasound devices and targeted scanning methods [112]. High-quality cardiac MRI (CMR) imaging necessitates precise patient positioning and skilled operators' preparation of image acquisition planes. For view-planning and anatomical localization of the heart, vendors have created ML-based automated software that has demonstrated excellent agreement with manual techniques [73,113,114]. AI has also been used to use CNN and random forest algorithms for the real-time detection and suppression of image artifacts [73,113,114]. Patients with HF and other CVDS can now have their long-term prognosis predicted thanks to recent developments in the application of AI to standard 12-lead ECGs. In an extensive regional health system, Raghunath et al. [115] employed 1,169,662 12-lead resting ECGs from 253,397 individuals over 34 years to create and verify a DNN model to predict 1-year all-cause death from ECG voltage-time traces.

The model's performance in forecasting one-year death remained strong (AUC = 0.85), even among the sizable subset of patients (n = 45,285) whose ECGs were judged by doctors to be "normal." This suggests that AI can significantly improve prognostic information in ECG interpretation [116]. ECG is frequently used to quantify left ventricular ejection fraction (LVEF), a crucial indicator of left ventricular systolic function [116,117]. Due to a slight drop in LVEF, patients with early-stage HF may exhibit asymptomatic left ventricular dysfunction (ALVD) for an extended period [116,117]. It is crucial to improve left ventricular systolic functions, prevent further LVEF decline and irreversible myocardial damage, increase survival rates, and enhance the quality of life for HF patients if they receive appropriate treatment [118]. However, due to availability and cost issues, echocardiography is impractical for asymptomatic patients [64,112]. AI-ECG has recently been shown in several trials to be useful for ALVD screening. Using the ECG and echocardiography data of 44,959 patients, Attia et al. [119] built a massive neural network to identify patients with cardiac dysfunction (ejection fraction [EF]≤ 35%) with ECG. The network achieved an accuracy of 85.7% and an AUC of 0.93 when tested on a group of 52,870 patients. Patients with positive AI-ECG but negative echocardiography (i.e., false positive) had a four-fold higher risk of developing left ventricular dysfunction (HR = 4.1) over a median follow-up of 3.4 years compared to those who were identified as having a normal EF by both the network and echocardiography (i.e., true negative). This suggests the network can locate abnormal ECG before left ventricular dysfunction manifests itself. In another similar study, Yao et al. [120] created an AI system to use the ECG to identify patients with low EF, defined as EF≤ 50%. One hundred twenty primary care teams from 45 hospitals and 22,641 individuals without HF were randomly assigned to the intervention or control groups. The AI-ECG results were available to the intervention group. According to this study, within 90 days following the ECG test, the identification of poor EF was improved by 32% when utilizing AI-ECG as opposed to the control group. Additionally, the application of AI-ECG in the outpatient context improved the diagnosis of low EF more (OR =1.71), suggesting that AI-ECG may facilitate the early detection of patients with low EF in primary care and resource-constrained settings [116]. AI methods may improve the effectiveness of auxiliary tools like MRI, CT, and echocardiography. LVEF is crucial in determining the prognosis of HF, resynchronization therapy, and defibrillator. An ML method that can reliably and automatically quantify LVEF was proposed by Asch et al. [121]. The accuracy of the ML in extracting LVEF was comparable to that of physicians. Clinical safety was enhanced by the nurses' ability to dynamically monitor patients' LVEF without needing a professional sonographer thanks to this well-trained, advanced algorithm. Moreover, the development of AI made it possible to scan and monitor echocardiograms for localized wall motion abnormalities [116]. With image segmentation accuracy ranging from 72 to 90 percent, Zhang et al. [64] trained a CNN that correctly detected views at 96% for the parasternal long axis or 84% accuracy overall. With good results (κ, 72.6%; 95% confidence range, 58.1-87.0), Sanchez-Martinez et al. evaluated velocity patterns to distinguish HF preserved ejection fraction (HFpEF) from healthy individuals. Similarly, Tabassian et al. [106] used strain parameters (asymptomatic vs symptomatic; AUC = 0.89; accuracy = 85%; sensitivity = 86%, specificity = 82%) to classify HFpEF patients with symptomatology phenotypes [64,106,122,123]. There have been reports of two different methods for using AI in cardiac imaging. Classical ML approaches are applied to various clinical and pre-computed picture attributes to predict diagnostic or prognostic outcomes from big datasets. Advanced AI techniques, such as deep learning techniques, have been used to diagnose real-life images. DL can be applied to outcome prediction or image-based disease identification [56]. In contrast to traditional AI methods, deep learning (DL) directly analyzes images for tasks like image segmentation or result prediction, eliminating the need for so-called "feature engineering," the calculation and extraction of "custom-tailored" imaging variables. Large, intricate datasets with plenty of features are ideally suited for deep learning (DL), such as those found in the imaging and genomics fields. The segmentation of cardiovascular images is a topic in development where AI-based techniques and intense learning techniques have seen significant performance improvements recently. Advanced imaging, modeling, multimodality registration, heart anatomical features segmentation, and real-time guidance are all necessary for interventional cardiology [124]. Clinical data variables, stress test variables, and DICOM image datasets (Digital Medicine) from SPECT (Single photon emission CT) myocardial perfusion image (MPI) scans (including gated, static, stress, and rest images) are all included in the REFINE SPECT (REgistry of Fast Myocardial Perfusion Imaging with NExt generation SPECT) [124].

In cardiovascular imaging, CMR imaging has become a vital tool over the last few years [122]. Significant technological advancements have improved CMR's capacity for risk assessment and diagnosis. for noninvasive left ventricular volume and ejection fraction evaluation CMR is considered superior [125]. It also makes tissue characterization possible to determine medical management. Strain is a newly discovered biomarker that, like echocardiography, can aid in the ascent of CMR. However, several CMR procedures, such as volume measurement and contour tracing, require significant time. By integrating ML architectures, CMR can reach new heights and explore uncharted territory in cardiovascular imaging [73,125-128].

Issues about scheduling, efficacy, and missed diagnosis arise throughout imaging. At every stage of image collecting, interpretation, and decision-making, the deployment of AI may save costs and increase value. Finally, besides the features retrieved from the pictures, the physician's final clinical diagnosis typically necessitates considering additional clinical information, such as age, patient history, and symptoms. Doctors complete this difficult "ad hoc" task frequently without using explicit probabilistic techniques [56]. ML techniques, which integrate imaging and clinical factors, may enable a quick and accurate calculation of post-imaging illness or outcome likelihood. Numerous recent studies have shown the effectiveness of this strategy, most notably SPECT MPI, where image analysis is highly automated compared to other modalities.

Future application and wearables

Wearables in HF

Despite the availability of life-saving treatment for HF with a reduced ejection fraction, there remains a significant discrepancy in the utilization and dosage of medication by established guidelines [129]. The progression and acceptance of digital health technologies and mobile health (mHealth) devices have the potential to tackle these problems. Wearables allow daily gathering of functional or physiological data outside the hospital [130]. The field of digital health is rapidly growing [131]. The integration of technology into healthcare is augmented by versatile data, the development of new software platforms, and the utilization of AI [132]. Digital health includes using information and communications technology, which includes advanced computing science and data analytics, to enhance health.

On the other hand, mHealth is a specific category under digital health that employs mobile and wireless technologies [133]. Wearable health devices are a mHealth component that can enhance HF care. Wearable devices are externally applied devices that record functional and physiological data to monitor or enhance health. 

HF is prevalent, particularly among Americans who are 65 years old [134]. Smartphone usage is prevalent among Americans, with 77% of the population owning a smartphone. However, this trend is less popular among older individuals, with only 46% of Americans aged 65 and over owning a smartphone [135]. Gaining insights into the acceptance of smartphones and other wearable devices for commercial use, as well as incorporating these devices into the practice of human factors (HF) clinical medicine, is crucial for advancing medical wearables. 

Smartphone sensors can utilize cardio ballistics, heart vibrations, and photoplethysmographic imaging of jugular venous pressure to monitor patients with HF [136]. Ultra-low-power skin patches, watches, and contact lenses can biochemically monitor lactate or electrolytes [137,138]. Fundus photography can be employed to observe physiological alterations in the retina of cardiovascular patients, such as those with HF [139]. The bathroom mirror has optical sensors that may evaluate cardiac hemodynamics by analyzing changes in skin color, pulsation, and pulse-wave velocity. These sensors are connected to other "smart" devices [130]. 

Types of devices

A great number of wearable gadgets are currently available in medicine. Smart watches, intelligent bands, earbuds, eyeglasses, innovative accessories, chest straps, smart rings, and smartphones are some examples of wearable technologies that are frequently utilized. In addition, additional sensor devices are incorporated into an individual's clothing.

Telemedicine, telemonitoring, mHealth, and eHealth have incrementally made their way into the realm of clinical practice over the past decade [140,141]. Continuous or occasional monitoring, which can be either dependent on the actions of the patients or entirely independent and automated, are the two types of monitoring included in telemonitoring [142,143]. These days, more advanced non-invasive systems can measure and transfer data that is measured non-invasively on electrocardiographic (ECG) tracings, oxygen saturation, blood pressure, and physical activity (e.g., a pedometer) [144]. In addition, these systems can measure and transfer data that are measured invasively by utilizing implantable devices, which enables the transfer of variables, such as impedance analysis and pulmonary artery or left atrial pressures [145,146]. The term "telemonitoring" can also be broken down into two categories: active and passive telemonitoring. Active telemonitoring through non-invasive devices requires the patient to take some action (e.g., making a video call) or to perform some kind of self-measurement (e.g., taking their blood pressure). Passive telemonitoring is an example of invasive implantable devices that send data to the receiving physician intermittently or continuously. It has recently been proven that implantable telemonitoring devices for multi-parameters or cardiac hemodynamic activity monitoring are an effective strategy to prevent frequent hospitalizations [147-149]. This position was established in recent years. It is still up for contention whether or not non-invasive approaches should be used for the remote monitoring of HF patients [145,150].

**Figure 4:**
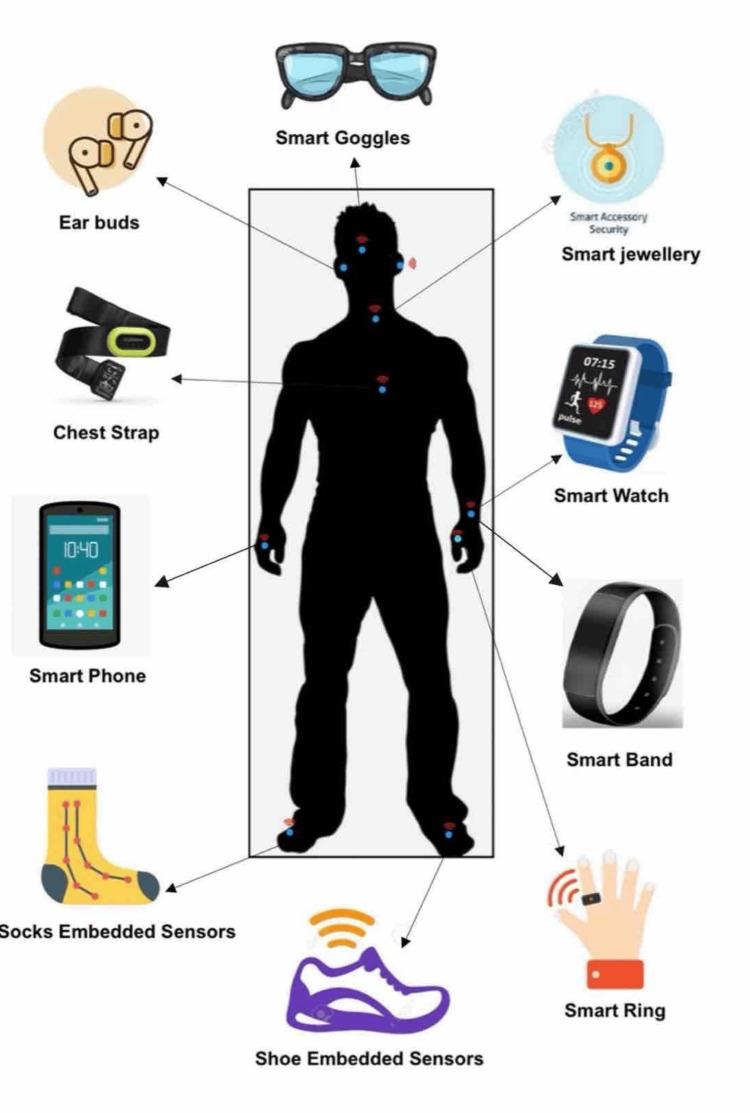
Types of devices used Source reference: [151] Figure made using Canva.com

Figure 4 represents the types of devices that could be used.

Accelerometer

**Figure 5:**
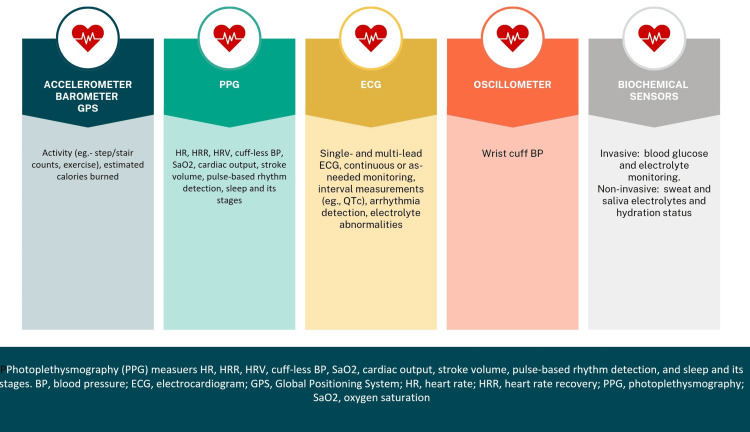
Sensors and measurements Source reference: [151,158,159] HR: heart rate, HRR: heart rate recovery, HRV: heart rate variability, GPS: global positioning system, SpO_2_: saturation of oxygen, ECG: electrocardiogram

There were 149 patients who were outfitted with gadgets by researchers from the Mayo Clinic [130]. Of these patients, 22 patients who had HF wore a Fitbit One (San Francisco, California), which is an accelerometer that is available for purchase and estimates step counts to evaluate mobility following successful cardiac surgery [130,152]. The results of the study showed that postoperative mobility could be monitored wirelessly in a hospitalized senior population, including those with HF [130]. In addition, the study found that patients who stayed for shorter or intermediate lengths of time were more ambulatory than those who stayed for longer periods of time [130]. In a similar manner, researchers from Denmark gave patients who were participating in a substudy of Teledi@Log (Telerehabilitation of Heart Patients; NCT01752192), a randomized-controlled trial of a telerehabilitation intervention in hospitalized cardiac patients, a gadget that was analogous to the Fitbit Zip [153]. The results of that substudy revealed that a telerehabilitation program is capable of incorporating step count data into the intervention [153]. In the NEAT-HFpEF (Nitrate's Effects on Activity Tolerance in Heart Failure With Preserved Ejection Fraction) trial, researchers from the Heart Failure Clinical Research Network of the National Heart, Lung, and Blood Institute of the United States used daily activity measurements that were evaluated by accelerometers that were worn by patients as the primary endpoint [154]. Utilizing a belt-worn activity monitor (Kersh Health, Plano, Texas) that featured accelerometers (KXUD9-2050, Kionix, Ithaca, New York), the researchers in that study compared the daily activity levels of patients with HF who had preserved ejection fraction while taking isosorbide mononitrate to those of patients who were given a placebo. The SenseWear Pro3 Armband (BodyMedia, Inc., Pittsburgh, Pennsylvania) and the Zephyr BioHarness and BioPatch (Medtronic, Dublin, Ireland) are two examples of accelerometer devices that have been utilized in research pertaining to the study of activity and step count [155,156]. AliveCor, a small monitor with two metal electrodes that create a single-channel, bipolar lead that transmits to a mobile application, is one example of a handheld ECG recording device that has been used to screen patients with HF for atrial fibrillation (AF) [157]. Handheld ECG recording devices have also been used to screen for AF [157]. A community-based screening program was conducted on 13,122 Hong Kong inhabitants, and the results showed that 239 of them had AF [157]. Of these, 97 citizens had HF, and it was discovered that HF was independently related to AF [157]. Sensor and measurements are depicted in Figure 5.

Smartwatches

The non-invasive method known as photoplethysmography, which employs a light source and a photodetector at the skin's surface to evaluate variations in blood circulation, is currently being utilized by smartwatches to identify abnormal rhythms [130]. This particular technology is frequently utilized to monitor heart rate, and the accuracy of the results may be affected by factors related to the patient (such as movement or ectopic beats) and environmental factors (such as ambient light or temperature) [130]. Accelerometer technology is now included in the Kardia Band, an accessory for the Apple Watch certified by the Food and Drug Administration (FDA) [160]. The Kardia Band was manufactured by Apple in Cupertino, California, and features accelerometer technology [160]. The precision of these devices is currently being investigated, and there needs to be more data about individuals with HF [160]. Kardia Band readings were compared to electrocardiograms both before and after cardioversion in research that had 100 consecutive patients with AF who presented for scheduled cardioversion [160]. However, the automated Kardia Band algorithm regarded 34% of the signals as unclassified, even though the Kardia Band had excellent sensitivity and specificity for diagnosing AF. Right now, the FDA has approved a smartwatch for monitoring blood pressure on the wrist [160]. Oscillometric measures are taken with the help of a cuff that is attached to the watch strap. In addition, the watch can wirelessly record measurements in an application [160].

HF decompensation can be predicted by using textile-based sensors (NCT03719079) [161]. With an accelerometer and stretch sensor embedded right in, a prototype of a "smart" sock has finally been produced to detect edema and activity [161]. This demonstrated a good level of accuracy when measuring the size of the ankle and the amount of physical activity in healthy adults [161].

The use of a Holter monitor was formerly necessary for ambulatory electrocardiogram monitoring; however, using a patch worn on the body is now possible [162]. Two such devices, the CarnationTM patch and the VivaLNKTM, have recently been approved by the FDA. These devices can be worn for several days at a time, providing continuous ECG monitoring that is more comfortable and lasts longer than a typical Holter monitor [162]. There is also the possibility of weaving electrodes into a "smart textile" vest for sustained multi-lead ECG monitoring, such as the CardioskinTM vest. PPG can, therefore, be used in conjunction with ECG monitoring using wearables to diagnose AF [162].

Pill Sensor

A pill that contains aripiprazole and an ingestible sensor that communicates with a cutaneous wearable to track adherence was recently approved by the FDA as an aripiprazole (antipsychotic) tablet with a sensor [163]. This tablet is called Abilify MyCite and was manufactured by Otsuka American Pharmaceutical in Rockville, Maryland [163]. The sensor transmits A signal to an abdominal cutaneous patch, which is engaged when the pill touches the stomach [163]. The data regarding adherence are subsequently monitored through a mobile application. It is possible to make use of this data in a variety of different ways in order to encourage medication adherence and to establish an environment to facilitate health coaching [163].

Pill Box Reminder

As an illustration, the pilot study that investigated a device known as MedSentry, an electronic pillbox that is remotely monitored and alerts patients when it is time to take their medication and connects patients with carers in the event that the medication is not taken, demonstrated a reduction in the number of hospitalizations for all causes and the length of stay for all causes in the intervention group [163].

Mobile Application

The Medly mobile phone application lets patients record their daily parameters, such as blood pressure, body weight, and symptoms [163]. Ware et al. [163] conducted another study that took a different approach to telemonitoring [163]. This study was more than just another study [163]. Patients receive automated phone calls to encourage them to participate in the study [163]. If the patients had not taken their readings by 10 AM, the call was sent to them. Medly's algorithms can generate self-care messages and clinician warnings based on patient-driven target ranges [163]. These messages are generated by evaluating the user as they submit information. The trial's objective was to incorporate Medly into the standard of treatment provided to patients with HF [163].

Using handheld ECG recording devices (AliveCor device, San Francisco, CA) that transmit to a smartphone application, Chan et al. screened patients with HF for AF [157]. This demonstrated the feasibility of the screening process and allowed for the identification of a significant proportion of patients who had recently been diagnosed with AF for the first time [157].

The detection of cardiac decompensation events is being made possible by the development of additional technologies, such as vests that measure the amount of fluid in the thoracic cavity and ballistocardiogram or seismocardiogram devices that measure whole-body motions and chest wall vibrations [164,165]. While there is a possibility that wearables could improve care and outcomes for individuals suffering from HF, the data that are currently available are restricted to feasibility studies and small randomized controlled trials [130].

ReDS

Sensible Medical Innovations (Kfar Netter, Israel) has developed the Remote Dielectric Sensing (ReDS) system to monitor the volume of lung fluid in patients who have HF [166]. This system consists of a wearable vest that contains two sensors that emit and intercept low-power electromagnetic signals that reflect the dielectric properties of tissue and the fluid content [166]. Because water has a high dielectric coefficient and air has a low dielectric coefficient, it is possible to determine the amount of fluid in the intrathoracic cavity [166]. Amir et al. [164] conducted a multicenter, prospective, single-arm trial in which they evaluated the ReDS system as an addition to the standard of care in a group of 50 patients who were being discharged after being diagnosed with acute decompensated heart failure (ADHF) [164]. At the time of their initial admission, patients were enrolled and monitored at home with daily assessments for 90 days. According to the findings, the implementation of ReDS-guided management led to an 87% reduction in readmissions compared to the three months prior to the intervention of ReDS [164]. After the vest intervention was discontinued, there was a 79% rise in readmissions [164]. A technique for determining whether or not a patient is ready to be discharged has also been utilized. As a result of the vest, Bensimhon et al. evaluated 110 patients who were considered ready for hospital release [167]. They discovered that 32% of patients had considerable residual lung congestion [167]. These patients were given a one-to-one feedback session with a physician on the vest readings before they were discharged, and they also received a consultation with a specialist in HF; the results are still awaited [167]. The FDA has recently approved this vest, which is now accessible for commercial use; however, additional research is required to substantiate these first positive results [167].

Bioimpedance Monitoring

Bioimpedance analysis is a method that may be utilized to determine body composition measurements by determining the amount of lean and fat body mass, total body water, and extracellular and intracellular water [167]. The relative change in the patient's fluid balance is significant in patients with HF, but a single measurement of the patient's fluid status could be more informative [167]. The reason for this is that an invasive method of obtaining a current that was created from the pacing wires of pacemakers and defibrillators was initially utilized to achieve a sensible method of analyzing the body water overload of the patients [168,169]. The fluid excess localized within the patient's thorax is taken into account by this type of measurement [168,169]. An example is the CRT-ICD device that Medtronic developed [170]. This device features an internal impedance meter that is referred to as Opti-Vol. The purpose of the DOT-HF experiment was to investigate whether Opti-Vol would impact the clinical outcomes of CHF patients [170]. This feature for bioimpedance analysis was examined as a part of the trial [170]. Comparing the intervention arm to the standard treatment arm, it has been found that it is related to a marginally statistically significant increase in the primary outcome, which is comprised of hospitalizations for HF and mortality from any cause [170].

*CardioMEMSTM* 

CardioMEMSTM is a wireless pulmonary artery pressure monitoring device approved by the FDA and marked with the CE certification [171]. It has been linked to a considerable reduction in the number of hospitalizations for HF and an improvement in quality of life [148,171,172]. It is based on the fundamental premise of monitoring the pressure in the pulmonary artery (PA) and detecting hemodynamic congestion at an early stage to reduce the requirement for hospitalization and increase the likelihood of survival [173]. During the operation of right cardiac catheterization, the PA sensor is positioned in a PA-accepted manuscript branch so that it remains there permanently. The width of the sensor is 3.5 millimeters, the thickness is 2 millimeters, and the length is 15 millimeters. Neither batteries nor additional cables are required to use it because it has a small wire bent at each end. The "CardioMEMSTM Heart Sensor Monitor Pressure to HaveImprove Outcomes in Heart Failure Patients NYHA Class III" (CHAMPION) experiment found that the CardioMEMSTM HF system was related to a reduction in the number of hospitalizations for HF [148,172]. After that, Desai et al. conducted a retrospective analysis of Medicare claims data and reported that the CardioMEMSTM implant resulted in a 45% reduction in the hospitalization rate for HF and a 31% reduction in hospitalization for all causes [174]. When it comes to the long-term care of chronic HF, CardioMEMSTM HF is a vital and cost-effective instrument. Furthermore, the use of the CardioMEMSTM device is hindered by a multitude of constraints. These limits include chronic renal disease with a reduced glomerular filtration rate (GFR) of less than 25%, chest circumference size greater than 65 inches, congenital heart disease, mechanical suitable heart valve, and other limitations.

Implantable Pulmonary Artery Pressure Measurement (Cordella™)

Although the CardioMEMSTM, mentioned earlier, is still the sole pressure measurement device approved by the FDA, additional modalities are now being evaluated. At the patient's residence, the CordellaTM system reports on the patient's overall health state [166]. In order to facilitate future review and management, the data that have been acquired might be distributed to healthcare providers [166]. To proactively deliver the information required to optimize patient care between office visits, the Cordella sensor incorporates data on the pressure of the PA into the Cordella system [166].

Improving blood pressure control 

One of the most prevalent causes or contributors to HF is hypertension, and hypotension is a common consequence of HF and the therapy for it. It is common for therapy modifications to be prompted by blood pressure readings, often from one-time examinations conducted at a clinic or during a home visit. Compared with manual measurement, the mean differences were -0.9 ± 6.8 mmHg for systolic blood pressure and -1.1 ± 5.5 mmHg for diastolic blood pressure [175]. At least one miniature smartwatch integrated sphygmomanometer, OmronTM HeartGuide, has been found to meet the criteria set forth by the American National Standards Institute to measure blood pressure by oscillometry across a range of blood pressures. It is possible that such a wearable device could assist in the optimal adjustment of antihypertensive or HF medicine, monitoring of iatrogenic hypotension, and encouraging persistence with therapy; nevertheless, the demonstration of such impacts is still under progress [175].

Technology in the development for early detection of HF decompensation

A clinical trial for predicting HF decompensation is now being conducted to test the ZOLL μCor™, which is a patch that has been approved by the FDA and is equipped with an ECG monitor, a radiofrequency sensor, and a transmitter. The patch is designed to quantify the amount of fluid in the pulmonary system. There is a correlation between the movement of the heart in the chest and the flow of blood through the arterial tree, and seismocardiography is the detection of chest wall vibrations that correlate with this heart movement [176]. Graphic analysis differentiated between compensated and decompensated patients' responses to a six-minute walk test in an observational study that included 45 HF patients wearing a seismocardiography patch [177]. In addition, within-subject improvements were found to correlate with changes in seismocardiography indices. In addition, algorithms could forecast the left ventricular ejection percentage in real time by combining information from several seismocardiogram sensors into a wearable vest [178]. 

HeartLogic™ algorithm

Multisensor Chronic Evaluation in Ambulatory Heart Failure Patients (MultiSENSE) was the source of the data utilized in developing the HeartLogic algorithm. Lung impedance, activity, respiratory rate, volume, and heart sounds (s1, s3) were all elements that were incorporated into the sensor measurements. In order to provide clinicians with a warning when a patient's HF is getting worse, the changes in sensors were weighted and detected on a risk estimate. This resulted in a single composite score. HeartLogic found the NT-proBNP assessment at the beginning of the study to be improved. According to a retrospective review, the HeartLogic algorithm may also help detect the gradual worsening of HF and stratify the risk of HF decompensation [179,180]. 

Due to the significant frequency of readmissions, the elevated death rate, the low standard of living, and the considerable expenses incurred by the national healthcare system, extensive endeavors have been undertaken to determine the parameters and risk factors that can aid in forecasting and averting instances of deterioration and avoidable hospitalizations [181,182]. Several trials have established the efficacy of RM in improving life expectancy, quality of life, and reducing HF rehospitalizations [144,183]. An analysis combining data from five trials examined the effects of hemodynamic-guided therapy of HF in patients with symptomatic HF. There was a reduction of approximately 38% in the likelihood of hospitalizations specifically related to HF [184-188]. Over the past decade, various studies have attempted to evaluate the impact of telemedical interventions and telemonitoring programs on the death and rehospitalization rates of patients with HF.

One significant constraint is the availability of high-quality data for its practical application into AI to ensure accurate outcomes. In the presence of errors in the data fed into the algorithm, or when the AI system is trained using relativist and biased input data, the outcome has incorrect results [26]. This is known as the GIGO (garbage in, garbage out) process [26].

Bourazana et al. [26] explained that when using two different AI algorithms (ESC HFA-PEFF and H2FPEF) for the diagnosis of HFpEF, even though the scores by both of those overlapped in certain variables, one of the two (ESC HFA-PEFF) incorporated some additional parameters, consequentially leading to potential disagreements regarding which scoring system to employ in the diagnosis of HFpEF [26]. Several factors, including loading conditions and structural changes in the left ventricle, influence LVEF. Hence, even though LVEF does not directly contribute to myocardial contractility, it is misinterpreted as such [26]. Therefore, the reliance on LVEF in the existing AI models for HF classification is another example of the challenges in the availability of accurate and high-quality data [26]. 

Inconsistencies in data quality and biases in the datasets when using diverse data sources can result in skewed AI models that eventually become unreliable tools for diagnosing HF [26,188,189].

Interpretability and explainability of AI models

AI models, especially those implementing deep learning, present significant challenges due to their “black-box" nature [190]. The term “black box" refers to the opaque nature of the internal working processes of their learning and decision-making functions [190,191]. Despite the possibility of having precise knowledge about the input data, the lack of transparency in understanding how the thinking process of the AI system led to the outcome makes it impossible to fully understand and rectify any mistakes that take place [191,192].

This also affects the clinician’s trust and willingness to accept the results, produced due to the lack of detailed explanations and the inability to navigate the decision-making process of the DL models. Moreover, there is a lack of insight for a clinician about the details of the statistical approximations being done due to a scarcity of technical and mathematical knowledge [190]. These problems surrounding interpretability and explainability engender ethical and legal quandaries as well.

Ethical and legal implications

The absence of transparency in such circumstances may entail legal ramifications as well since patients can ask to be properly informed and explained regarding the utilization of their personal data [190]. Gonzalez-Alday et al. [190] mentioned the GDPR (General Data Protection Regulation) as one of the latest examples of legal regulations about this matter, wherein the European Union has started to integrate the requirement of transparency of AI where there is sensitive data in use [190].

Furthermore, the question of legal and ethical consequences also arises from the fact that there are chances of misdiagnoses [190-193]. AI may interpret various correlated factors as causation without fully understanding the reasons behind their relationship, basically overlooking important connections or mistakenly attributing factors as causation [190-193].

There is a notable compromise between the efficacy of an AI model and its capacity to generate transparent and comprehensible predictions [194]. Apart from the potential of the black-box models leading to errors and eventually hazardous decisions, there is a danger of the possibility of influencing the system by malicious actors, through manipulation of the input data to alter the outcomes [194].

Generalizability across diverse patient populations

Despite providing high-quality input data and facilitating transparency and straightforward interpretability, another critical challenge is generalizing the findings across heterogeneous patient cohorts [18]. Insufficient data exist on congenital heart diseases stratified by race/ ethnicity, which causes doubt about the adequate representation of all populations due to the lack of equitable availability of datasets encompassing diverse racial and ethnic groups [18,195,196]. Moreover, there is the concern of the exclusion of entire countries from research due to limited financial and research resources [195,196].

One such example of underrepresentation is shown by Bayne et al. [195], where it is mentioned that in the USA, Black and Hispanic individuals are significantly underrepresented in clinical trials, with participation rates as low as 5% and 1% [195]. In another instance, a 2018 study revealed that three facial recognition platforms recognized by law enforcement agencies showed significant discrepancies, as 35% of dark-skinned women were mistakenly identified as men. By contrast, there were minimal errors for light-skinned men [196]. To summarize, it is clear that datasets derived from mostly homogenous populations perpetuate biases when applied to a diverse demographic [195-197].

Future study and challenges

The ever-evolving nature of technology and communication platforms (e.g., 4G versus 5G) presents developers with several obstacles, the most significant of which is the requirement for ongoing updates and modifications [198]. It is the responsibility of the developers of wearable devices to guarantee that the measures they produce are correct (e.g., reliable readings of the heart rate), which can be affected by movement and activity under certain circumstances. The fact that there is a steady flood of new companies and new technology makes it even more challenging to get data about patients and consumers. In the healthcare industry, developers are entering the market to sell consumer devices that assess essential indicators. These products may or may have yet to receive regulatory approval. As a result, this might lead to confusion and overload in the market, which can result in consumer weariness or bewilderment and restrict the ability to collect data from the actual world regarding the efficacy of interventions related to the utilization of digital health devices and methods [199].

The advent of wearable sensors in high frequency (HF) can potentially create a "digital divide." In addition to the clinical heterogeneity that is seen in patients who have HF, socioeconomic status (SES) plays a significant impact. Studies have shown that, with lower SES, there is a greater than 50% rise in the risk of HF [200,201]. The incorporation of device-related charges into healthcare insurance benefit packages is necessary in order to reduce the digital gap that is established based on economic disparities. In addition, loaner digital wearables have the potential to assist in offseting the financial effects on healthcare, provided that safeguards are in place to ensure that the digital gadgets are returned without any problems [202]. A lack of digital literacy can not only hinder the implementation of digital technology but also affect the degree to which patients comply with data transmission. It should come as no surprise that persons who have completed college or possess a higher level of education have been demonstrated to be more open to digital technology than those who have a lower level of education [203].

In addition, older adults are less likely to adopt digital technology and comply with regular data transmission [203]. This decreases the likelihood of adoption. Even while it is of the utmost importance to create methods that will enhance the utilization of wearables in this population at risk, the question of how these measures may be implemented in patients with neurocognitive disorders and visual/hearing impairments continues to be tough.

## Conclusions

The utilization of AI in cardiology holds vast potential, particularly in the diagnosis of HF, showcasing promising outcomes. Integrating AI with traditional diagnostic methods enables early detection of HF and offers numerous advantages to both healthcare systems and patients. These include expedited and precise diagnoses, even in resource-limited settings, and the potential to alleviate the financial burden associated with HF management. AI-driven approaches have made significant strides in HF detection, particularly in ECG analysis, advanced cardiac imaging, leveraging cardiac biomarkers, and cardiopulmonary stress testing. Its potential grows with the integration of AI from wearables to advanced medical devices. However, challenges such as access to high-quality data, model interpretability, ethical concerns, and generalizability across diverse populations remain. Nevertheless, ongoing efforts by experts to refine AI models indicate a promising future for diagnosing heart failure with AI technology.
